# Hepatitis C virus risk among young people who inject drugs

**DOI:** 10.3389/fpubh.2022.835836

**Published:** 2022-07-29

**Authors:** Pedro Mateu-Gelabert, Nasim S. Sabounchi, Honoria Guarino, Courtney Ciervo, Kellie Joseph, Benjamin J. Eckhardt, Chunki Fong, Shashi N. Kapadia, Terry T. K. Huang

**Affiliations:** ^1^Department of Community Health and Social Sciences, CUNY Graduate School of Public Health and Health Policy, Institute for Implementation Science in Population Health (ISPH), New York, NY, United States; ^2^Department of Health Policy and Management, Center for Systems and Community Design (CSCD), CUNY Graduate School of Public Health and Health Policy, New York, NY, United States; ^3^Department of Medicine, NYU School of Medicine, New York, NY, United States; ^4^Department of Population Health Sciences, Weill Cornell Medicine, New York, NY, United States

**Keywords:** hepatitis C virus, people who use drugs, people who inject drugs, systems thinking, qualitative system dynamics

## Abstract

**Background:**

Injection drug use (IDU) is the leading risk factor for hepatitis C virus (HCV) transmission in the U.S. While the general risk factors for HCV transmission are known, there is limited work on how these factors interact and impact young people who inject drugs (YPWID).

**Methods:**

Project data were drawn from a study of 539 New York City (NYC) residents ages 18-29 who were recruited *via* Respondent-Driven Sampling and, reported past-month non-medical use of prescription opioids and/or heroin. Analyses are based on a subsample of 337 (62%) who reported injecting any drug in the past 12 months. All variables were assessed *via* self-report, except HCV status, which was established *via* rapid antibody testing. Integrating the observed statistical associations with extant literature on HCV risk, we also developed a qualitative system dynamics (SD) model to use as a supplemental data visualization tool to explore plausible pathways and interactions among key risk and protective factors for HCV.

**Results:**

Results showed a 31% HCV antibody prevalence with an overall incidence of 10 per 100 person-years. HCV status was independently correlated with having shared cookers with two or more people (AOR = 2.17); injected drugs 4–6 years (AOR = 2.49) and 7 or more years (AOR = 4.95); lifetime homelessness (AOR = 2.52); and having been incarcerated two or more times (AOR = 1.99). These outcomes along with the extant literature on HCV risk were used to develop the qualitative SD model, which describes a causal hypothesis around non-linearities and feedback loop structures underlying the spread of HCV among YPWID.

**Conclusions:**

Despite ongoing harm reduction efforts, close to a third of YPWID in the community sample have been exposed to HCV, have risks for injection drug use, and face challenges with structural factors that may be preventing adequate intervention. The qualitative SD model explores these issues and contributes to a better understanding of how these various risk factors interact and what policies could potentially be effective in reducing HCV infections.

## Background

Injection drug use (IDU) is the leading risk factor for Hepatitis C Virus (HCV) transmission across urban, suburban, and rural settings in the United States (U.S.) (1). Recent national surveillance data reveal an increase in reported cases of acute HCV infection every year from 2011 through 2018, with incidence nearly tripling (1,232 reported cases in 2011 vs. 3,621 reported in 2018. For each year between 2008-2018, young adults (ages 20–29 years) had the highest rates of newly reported acute HCV infections, more than any other age group (2). Despite the fact that urban areas such as New York City (NYC) generally have a wider availability of harm reduction resources to reduce the incidence of HCV, the prevalence of HCV among young people who inject drugs (YPWID) is similar to that of suburban and rural areas (3). These similar prevalence might be driven by opioid injection as an independent factor associated with HCV positivity across a variety of urban and rural localities (e.g., Los Angeles, New York, Montreal, Appalachia) (3–7).

Now in its second decade, the opioid epidemic, with its intertwined use of prescription opioids (POs), heroin, and illicitly manufactured fentanyl, has led to an increase in IDU, continuing to fuel an increase in HCV infections (8, 9). For many young people, sustained non-medical use of POs has led to heroin use because heroin is more readily available and cheaper than POs (10–12). Data from the National Survey on Drug Use and Health (NSDUH) indicates that 4 out of 5 current heroin users started using POs beforehand (13). Many young people who experimented with non-medical opioid use and heroin have transitioned to IDU, putting them at risk of HCV exposure (7, 12, 14, 15).

Specific injection-related risk factors associated with HCV infection among people who inject drugs (PWID) include needle sharing, sharing cookers and other injection paraphernalia (16), length of injection career (5, 16, 17), injecting in public spaces (17), and injecting POs (4, 6, 7). Beyond injection risk behaviors, several researchers have reported on structural vulnerabilities that appear to be associated with HCV transmission among PWID including high rates of opioid misuse and drug overdose, unemployment, poverty (18, 19), incarceration (20–22), and homelessness (18, 23–25). It is likely that these factors interact, and act in concert with individual-level risk factors to promote HCV transmission and hinder access to testing and treatment, but these interactions are not well-studied.

A systems science approach, including the construction of qualitative causal models, can expand our ability to learn from evidence and design policies or interventions to address complex challenges without resulting in unintended consequences (26). SD modeling is a systems science methodology that is well suited for population studies where multiple feedback effects, time delays, and non-linearities are taken into account. SD has been used previously and is increasingly being applied to public health problems including chronic disease (27), infectious disease epidemics (28), HIV (29), and drug abuse (30). Recent studies have applied SD to explore the impact of potential policy changes, including changes in opioid prescription dosage, drug diversion, opioid use disorder (OUD) treatment, and naloxone distribution, on opioid-related outcomes (31–33). However, to our knowledge, no studies have used SD modeling to explore the spread of HCV among YPWID, despite the interdependence and non-linearity of multiple underlying factors.

SD modeling has both qualitative and quantitative aspects which both contribute to the understanding of complex dynamic systems and lead to policy insights (34, 35). Identifying feedback loops from qualitative causal maps can assist with explaining dynamic trends and forms the basis for developing the quantitative simulation model (34). In this article, informed by relevant HCV literature and an observational study of YPWID in NYC, we constructed a conceptualization of the HCV dynamics among YPWID using a qualitative system dynamics (SD) stock and flow diagram that allows for exploration of the complex structures and feedback loops underlying the HCV epidemic in this group. This analytic approach could be very useful to learn from the HCV epidemic and its growth among YPWID, which is influenced by several biological and behavioral factors as well as by the various domains of these factors (individual, interpersonal, community, and policy) and their interconnections (26).

## Methods

### Study design and procedures

This study presents selected findings from a larger investigation that assessed the drug use practices and health risks of young adults (ages 18–29) who used opioids (including non-medical use of POs and/or heroin use) in NYC. The current analyses focused on patterns and correlates of HCV infection among study participants who injected drugs; they were drawn from a larger sample of young opioid users. Quantitative data were used to establish the prevalence of HCV antibody-positive status among this subset who reported having injected drugs in the past twelve months. HCV prevalence by duration of IDU (in years) and incidence per 100 person-years were also estimated for this group.

Study participants were recruited using Respondent-Driven Sampling (RDS), a form of chain-referral sampling technique designed to engage hard-to-reach populations by using participants' personal network connections to drive recruitment. This method, which can reach people who may not frequently be found in street settings, may yield a more representative sample than street recruitment. Using this method, an initial set of 20 “seeds” was directly recruited by research staff through referrals from harm reduction services, drug treatment programs, participants in the study's formative qualitative component, and other research projects. Each participant completed a structured assessment and was invited to refer up to three eligible peers from their opioid-using contacts to participate in the study.

This process was repeated with the seeds' recruits and for successive sample waves, leading to a total of 539 participants enrolled from August 2014 to April 2016. Eligibility criteria included non-medical use of POs and/or heroin use 3 or more times in the past 30 days, current residence in NYC, 18–29 years old, English-speaking, and the ability to provide informed consent. Participants provided written informed consent and were compensated $60 USD for completing the assessment; an additional incentive was provided for each eligible participant they referred. Further details on the RDS methods are described elsewhere (36). The study was approved by NDRI's Institutional Review Board, and all participants provided written informed consent.

Study participants completed a computer-assisted, interviewer-administered structured assessment lasting 90–120 min and provided a fingerstick blood sample for on-site HCV antibody testing. The study instrument included sociodemographic and behavioral questions (951 questions organized in 27 sections) related to the following domains: substance use and drug injection history and current practices; injection-related HIV/HCV risk behavior; opioid use and injection networks; and lifetime and recent overdose experiences, among other topics. The present analyses in the study were based on this subset of the total sample (62%, *n* = 337/539) who reported both non-medical use of POs and/or heroin use 3 or more times in the past 30 days and injecting any drug for non-medical purposes at any point in the 12 months prior to the assessment. None of the participants who did not report injecting drugs tested HCV antibody positive (Ab+).

The study sample consisted of 337 opioid injectors, 65% men and 34% women. The mean age of the sample was 25 (SD = 3; range 18–29). Thirty-nine percent of the sample had completed high school or received their GED and 37% attended some college. The majority of the sample was White (74%), followed by Latinx (18%), and 8% other. Thirty-nine percent reported an annual household income while growing up of $50,000 or less; 35% from $51,000–$100,000 and 26% reported incomes while growing up of $101,000 or more. Sixty-nine percent had experienced homelessness during their lifetime.

Thirty-nine percent reported receptive syringe sharing in the 12 months before participating in the study: 26% with one person and 13% with 2 or more people. Sixty percent reported sharing cookers in the past 12 months: 21% shared cookers with one person and 39% shared with 2 or more people. A full description of this cohort can be found in our previous publications (12, 37).

### Measures

In order to gain a better understanding of what variables were associated with HCV Ab prevalence among young people who inject drugs in NYC, informed by the literature, we chose behavioral (e.g., sharing practices and years of IDU) and upstream variables (e.g., incarceration and homelessness). In structured assessments and statistical analyses, non-medical use of POs was defined as the use of POs “not prescribed for the respondent or use of these drugs only for the experience or feeling they caused” (38). The injection of POs was defined as injecting any opioid intended for oral intake. Two injection risk variables were assessed, sharing syringes and sharing cookers, both measured by the number of people with whom the sharing took place in the 12 months prior to the structured assessment. Sharing syringes was defined as using a syringe that had been previously used by someone else. Sharing cookers was defined as using a cooker someone else had previously used or using it simultaneously with someone else.

Years of IDU for each participant were determined by first calculating the number of days from the reported date of first injection to the date of the interview. For date of first injection, participants were asked to report the month and year of their first injection. The 15th of the reported month was used as the default for first day of injection. The number of days resulting from subtracting date of interview to date of first injection (the 15th of the month reported), was divided by 30.4375 and rounded to the closest integer to obtain the number of months each participant had injected drugs. The conversion from months to years of IDU for 0 and 1 year was as follows: 0–11 months = 0 years; 12–18 months = 1 year. Additional years, from months of IDU, were determined as follows: 19–30 months = 2 years; 31–42 months = 3 years; 43–54 months = 4 years; 55-66 months = 5 year; 67–78 months = 6 years; 79-90 months = 7 years; more than 91 months = 8 years or more.

Incarceration was measured by the number of times a participant was in jail or prison, independent of the duration of the detention or sentence. Homelessness was defined as staying on the street, in a shelter, in a Single Room Occupancy hotel (SRO), temporarily staying with friends or relatives, or living in a car. All variables were based on self-report data except for HCV antibody status, which was assessed with point-of-care rapid testing using the OraQuick Advance Rapid HCV Antibody Test (OraSure Technologies, Inc., Bethlehem, PA).

### HCV prevalence and incidence by years of IDU

HCV prevalence by years of IDU was calculated by dividing the number of HCV Ab+ participants with a given year(s) of IDU (i.e., 1 year, 2 years…) by the total number of participants with those given years of IDU. Given that it is impossible to pinpoint the date of HCV exposure among this community sample, incidence calculations rely on several assumptions similar to those outlined by Jordan et al. in the study of HCV incidence in NYC between 2006-2013 (39): a) all participants were HCV negative when they started injecting; b) HCV Ab+ participants seroconverted at the midpoint between first injection and time of study interview; and c) those who reported injecting less than a year were assumed to have injected for 0.5 year (39). HCV incidence was calculated by a formula in which the numerator included the total number of HCV Ab+ participants with a given number of years of IDU (i.e., 1 year, 2 years …). The denominator equaled the total number of IDU years for HCV antibody-negative participants plus half the total years of IDU for HCV Ab+ participants. The result was multiplied by 100 (and rounded to the nearest integer) to obtain HCV incidence per 100 person-years.

Prevalence and incidence data are based on 332 of the 337 YPWID for whom we could ascertain years of IDU. Thirty percent (n=101) of YPWID tested HCV antibody-positive. The sample HCV antibody-positive prevalence and incidence per years of IDU (from less than a year to 8 years or more) are presented in Table 1. The prevalence of HCV among those who had injected for <1year was 2.9%; this increased to 12.5% among those who had injected for 1 year, 20.9% among those who had injected for 2 years, and 28.9% among those who had injected for 3 years. Prevalence was between 40–48% for those who had injected for 5 to 7 years. The highest prevalence was among those who injected for 8 years or more (59.7%). The incidence per 100 person-years of IDU (PYI) was 6 for those who had injected less than a year and more than doubled to 13/100 PYI for those who had injected for 1 year. Incidence remained near 10/100 PYI (range 7–12) for the subsequent 2–7 years of IDU.

**Table 1:** Hepatitis C virus (HCV) antibody status by years of injection drug use: prevalence and incidence per 100-person years (*n* = 332*).

				**Sample**	**Incidence**
Years of Injection		HCV-	HCV+	Prevalence	per 100 person years
<1 year	(1–11 montds)	68	2	2.9%	6
1 year	(12–18 montds)	7	1	12.5%	13
2 years	(19–30 montds)	38	10	20.8%	12
3 years	(31–42 montds)	27	11	28.9%	11
4 years	(43–54 montds)	20	6	23.1%	7
5 years	(55–66 montds)	19	14	42.4%	11
6 years	(67–78 montds)	18	12	40.0%	8
7 years	(79–90 montds)	9	8	47.1%	9
8 yrs or more	(> = 91 montds)	25	37	59.7%	
	Total	231	101	30.4%	10

*Sample total is 332 instead of 337 because we could not ascertain years of injection for 5 participants.

### Data analysis

#### Statistical approach

All statistical analyses were conducted in R, versions 3.2.2 and 3.2.4 (R: A language and environment for statistical computing. R Foundation for Statistical Computing, Vienna, Austria, http://www.R-project.org/) and IBM SPSS v.25 (Chicago, IL). First, binary associations of the variable of interest (HCV antibody-positive status) were computed with a series of sociodemographic (gender, race/ethnicity, household income growing up, lifetime homelessness) and injection risk variables (syringe- and cooker-sharing in the past 12 months), as well as number of incarcerations, years of IDU, injecting POs, and knowing one or more opioid users older than 29 years old. Log ratios and *p*-values were computed for all binary associations using a Wald chi-squared test, with a 95% confidence interval (40). Following the strategy described in Hosmer et al., variables with *p* < 0.25 in bivariate analyses were then included in a multivariable model (41). The multivariable model was run using a generalized linear model (R version 3.2.4, glm 4.13-19), and adjusted odds ratios were computed using the model estimate. Results were verified with logistic regression in SPSS.

#### Qualitative system dynamics (SD)

Although our statistical analyses identified significant linear associations between the measures describe above with HCV infection, they do not paint a causal picture that fully reflects the interactions and dependencies between these variables. Significant bivariate or multivariable associations can provide initial insight into factors correlated with the complexity of how HCV is spread, but these associations represent linear thinking; they are independent of each other, and do not indicate differences in degree among different associations. The depiction of actual decision-making processes of actors within the system is also omitted (42, 43). In order to study complex problems such as the HCV epidemic, we needed to expand the model to include how the structure of the system and interactions among its elements determine HCV outcomes. In using SD modeling to establish a framework (causal diagram) to examine the HCV infection dynamics in YPWID, we identify the structure of a complex system that drives the epidemic. We explicitly depict, through this modeling, the interdependencies and feedback loops between causal variables and epidemiologically important outcomes (42).

Evidence that there is a positive or negative association between two variables can be used to support linking two variables in a SD model. This is not sufficient evidence of causality, but still provides some support for the existence of causal influence of the antecedent variable on the outcome. Building on the statistical results in combination with previous publications from our research group and knowledge from extant research on HCV transmission, we developed a qualitative model using SD methodology (43) in the Stella? Architect modeling software (44). The model serves as a supplemental data visualization tool to depict a hypothesized dynamic structure and sequence of actions that impacted and led to changes in the number of HCV infections among YPWID. This exploratory qualitative SD approach attempted to integrate key structural, behavioral, and biological factors into a single framework that demonstrates the underlying causal feedback loop structures. The iterative process of SD model building (45) generates confidence in support of the hypothesized feedback loops that define the problem of focus.

## Results

### Bivariate and multivariable associations

In consideration of the purpose and scope of the problem, key variables were chosen to test their association with HCV Ab+ status. Bivariate and multivariable results are displayed in Table 2. In the adjusted model, there were significantly higher odds of HCV positive status among those who: had experienced lifetime homelessness [adjusted odds ratio (AOR): 2.52, 95% confidence interval (CI): 1.19–5.36]; shared cookers with two or more people in the past 12 months (AOR: 2.15, 95% CI: 1.05–4.37); had been incarcerated 2 or more times (AOR: 1.99, 95% CI: 1.06-3.73); and had injected drugs for 4 to 6 years (AOR: 2.49, 95% CI: 1.22–5.09) or for 7 or more years (AOR: 4.95, 95% CI: 2.35–10.46).

**Table 2:** Correlates of hepatitis c virus (HCV) antibody-positive serostatus among young people who inject drugs (PWID) (*n* = 337).

	**HCV–**	**HCV**+	**Unadjusted OR (95% CI)**	**OR** ***p*****-value**	**AOR (95% CI)**	**AOR** ***p*****-value**
*n* (%)	234 (69)	103 (31)	—	—	—	—
Gender						
Male	152 (66)	68 (67)	Ref	Ref		
Female	79 (34)	34 (33)	0.96 (0.59–1.58)	0.878	—	—
Race/Ethnicity						
Latino/a	43 (19)	18 (17)	Ref	Ref		
White	171 (74)	77 (75)	1.08 (0.58–1.99)	0.815	—	—
Non–Latino/Non–white	18 (8)	8 (8)	1.06 (0.39–2.88)	0.906	—	—
Household income growing up (annual)*				
$0–50,000	85 (38)	36 (42)	Ref	Ref		
$51–100,000	73 (33)	35 (41)	1.13 (0.65–1.98)	0.665	—	—
> $100,000	64 (29)	15 (17)	0.55 (0.28–1.10)	0.090	—	—
Homeless (lifetime)						
No	91 (39)	11 (11)	Ref	Ref	Ref	Ref
Yes	143 (61)	91 (89)	5.26 (2.67–10.38)	<0.01	2.52 (1.19–5.36)	0.016
Injected POs (lifetime)						
No	106 (46)	20 (20)	Ref	Ref	Ref	Ref
Yes	126 (54)	79 (80)	3.32 (1.91–5.79)	<0.01	1.25 (0.63–2.48)	0.517
Number of people shared syringes with (past 12 months)			
0	156 (67)	48 (47)	Ref	Ref	Ref	Ref
1	59 (25)	28 (27)	1.54 (0.89–2.68)	0.125	1.43 (0.71–2.86)	0.317
2 or more	18 (8)	27 (26)	4.88 (2.47–9.61)	<0.01	2.17 (0.91–5.16)	0.080
Number of people shared cookers with (past 12 months)			
0	109 (47)	26 (25)	Ref	Ref	Ref	Ref
1	53 (23)	17 (17)	1.32 (0.66–2.64)	0.433	1.35 (0.57–3.18)	0.491
2 or more	70 (30)	60 (58)	3.59 (2.07–6.22)	<0.01	2.15 (1.05–4.37)	0.035
Number of times incarcerated (lifetime)				
None	108 (46)	28 (27)	Ref	Ref	Ref	Ref
1	53 (23)	10 (10)	0.73 (0.33–1.61)	0.432	0.49 (0.19–1.22)	0.128
2 or more	72 (31)	64 (63)	3.43 (2.01–5.85)	<0.01	1.99 (1.06–3.73)	0.032
Number of years Injected drugs				
0–3	140 (61)	24 (24)	Ref	Ref	Ref	Ref
4–6	57 (25)	32 (32)	3.27 (1.78–6.04)	<0.01	2.49 (1.22–5.09)	0.012
7+	34 (15)	45 (45)	7.72 (4.15–14.37)	<0.01	4.95 (2.35–10.46)	<0.01
Know one or more opioid user(s) older than 29			
No	127 (54)	43 (42)	Ref	Ref	Ref	Ref
Yes	107 (46)	60 (58)	1.66 (1.04–2.65)	0.035	1.04 (0.58–1.86)	0.889

*29 (9%) participants did not respond to this household income question.

*AOR, Adjusted Odds Ratio; OR, Odds Ratio; POs, Prescription Opioids*.

*Bold values denote statistical significance at the p <0.05 level*.

### Qualitative conceptualization of HCV dynamics using a SD stock and flow diagram

In extending the statistical inferences and findings from our study sample, we transitioned to a qualitative SD model to visualize how the system impacting HCV outcomes among YPWID operates. We developed the model, drawing on findings from this study sample (6, 37, 46–52) and previously published literature on HCV transmission among YPWID (5, 16, 17).

According to the bivariate and multivariable statistical analyses, homelessness, PO injection, having shared syringes and cookers with 2 or more people, having IDU for 4 or more years, having been incarcerated 2 or more times, and having known any opioid users older than 29 years of age, were associated with HCV antibody-positive status. Although these associations provide some initial insights into the complexity of how HCV is spread, in order to study complex problems such as the HCV epidemic, the model was expanded to include how the structure of the system and interactions among its elements determine HCV outcomes. In the SD model, we identified the structure of the system by depicting different feedback loops that visualize causal processes (42).

**Figure 1:**
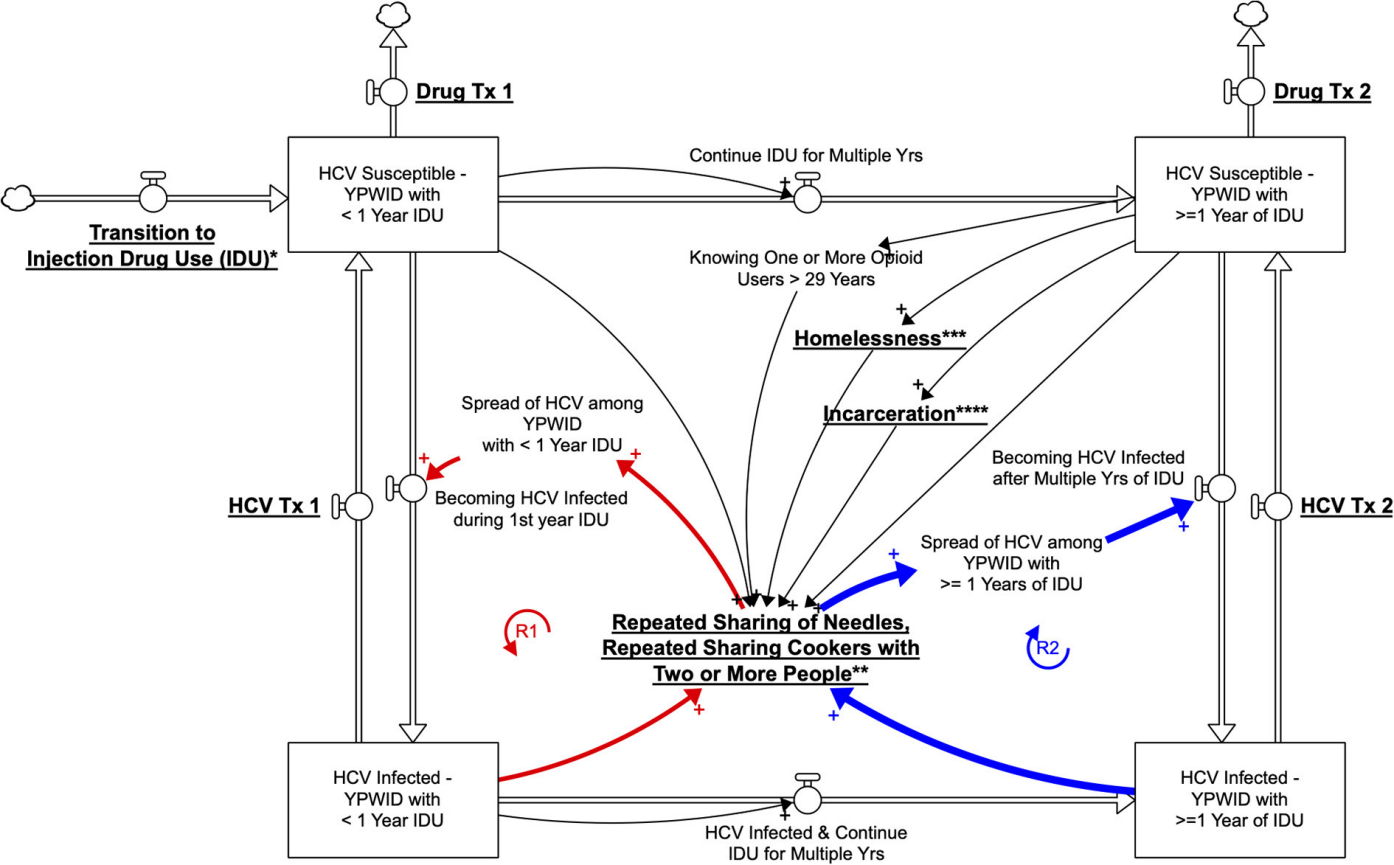
Qualitative System Dynamics Stock and Flow Diagram Visualizing HCV Dynamics among YWPID (*policy/intervention leverage points are underlined and bolded;* *Prevention of Transition to IDU, **Harm Reduction Services, ***Housing Policies, ****Reducing Incarceration).

We built the SD model by developing a stock-and-flow diagram that visualizes the physics and operations of the system impacting HCV outcomes among YPWID (see Figure 1). In this stock-and-flow diagram, we defined different stock (or state) variables as represented by boxes to capture a significant dynamic at the individual level, a vulnerability to HCV infection corresponding to years of IDU. We opted to present different stages in the drug injection trajectory and HCV infection status of YPWID represented by the following stocks: “*HCV Susceptible—YPWID with*<*1 Year of IDU*,” “*HCV Infected—YPWID with*<*1 Year of IDU”*, “*HCV Susceptible—YPWID with* ≥*1 Years of IDU”*, and “*HCV Infected—YPWID with* ≥*1 Years of IDU”*. Each stock connects to inflow and outflow arrows that represent the flow of individuals coming in and out of the system or transitioning from one state to another. For example, when “*HCV Susceptible—YPWID with*<*1 Year of IDU*” become HCV infected during their first year of IDU, they flow to the stock at the lower left-hand corner of “*HCV Infected—YPWID with*<*1 Year of IDU*”. Similarly, as YPWID continue injecting drugs over multiple years, they become more vulnerable to HCV as the risk of infection increases per years of IDU (also see Table 1). Thus, they move to the stock of “*HCV Susceptible—YPWID with* ≥*1 Years of IDU*”.

The specific cutoff point of 1 year is based on the incidence findings presented earlier, which showed that HCV incidence among YPWID with <1 = year of IDU (6 per 100 PY) is approximately half that of YPWID with 1 year of IDU (13 per 100 PY). As years of IDU increase, YPWID are more likely to know (and to inject drugs with) older PWID who are more likely to be HCV infected, leading to a higher risk of infection (39, 50). Similarly, continued IDU among PWID over multiple years leads to an increased likelihood of sharing syringes and other injection paraphernalia with HCV-infected PWID. The greater the cumulative number of sharing events, the higher the likelihood of HCV infection for YPWID, hence their transition to the stock of “*HCV Infected—YPWID with* ≥*1 Years of IDU*”.

Figure 1 represents the hypothesized pathways over time between variables using arrows with a polarity sign and the resulting feedback loops relevant to the outcome of HCV exposure. The arrows are accompanied by a polarity sign, with the positive sign (+) indicating that two variables changed in the same direction. For example, an increase in “years of IDU” was associated with an increase in the number of “YPWID who are HCV Ab+.” Negative signs would indicate that two variables changed in opposite directions; for example, “harm reduction services” would reduce the “repeated sharing of needles, or cookers with two or more people.”

A closed sequence of arrows (i.e., complete circles) forms a feedback loop. In the model, we identify two major reinforcing (R) feedback loops as highlighted by bold arrows. A reinforcing loop has zero or an even number of negative links and can create virtuous or vicious cycles, leading to exponential growth or exponential decline, where a problem becomes better or worse over time, often at an increasing rate.

The reinforcing loops illustrated in Figure 1 provide some initial insights in regard to the possible acceleration or slowdown of the rate at which YPWID become infected with HCV. The reinforcing loops R1—*HCV Spread among YPWID with*<*1 Year of IDU* and R2—*HCV Spread among YPWID with* ≥*1 Years of IDU*, refer to the increase in HCV infections among YPWID who came in contact with YPWID already infected through repeated sharing of needles and cookers with two or more YPWID. This structure represents an adaptation of the classic contagion structure referred to as the *SIR: Susceptible-Infected-Recovered* model (53). The underlying key assumption is that the number of individuals who become exposed to HCV is a function of the number of persons who already have hepatitis C. Therefore, if more young opioid users transition to IDU, it is likely that they will come into contact with YPWID who have HCV and will also become infected, making this loop a vicious cycle. The exploratory qualitative model in Figure 1 graphically represents interactions over time among key variables and describes feedback structures that might govern the dynamics of IDU, HCV risk, and HCV prevalence among YPWID. For clarity, in the model (Figure 1), the text presenting policy/intervention leverage points is underlined and bolded.

## Discussion

The present study reports an overall HCV antibody prevalence of 30.4% for a community sample of young people ages 18-29 who injected drugs, recruited between 2014–2016 in NYC. This HCV prevalence is lower than the 42% in Lower East Side and 51% in Harlem reported by Diaz et al. between 1997-1998 (54) and the 45% in the Lower East Side reported by Eckhardt et al. between 2005–2012 (17) among similar samples of street-recruited YPWID. The lower prevalence reported in the present study might indicate a decrease in the overall HCV prevalence among YPWID in NYC. This possible overall trend has also been documented in the NYC Hepatitis A, B, and C annual report, 2018, which indicated a 22% reduction in the number of HCV chronic infections among those 29 or younger during the period 2009-2018 (55). The overall HCV incidence among the study sample was 10 per 100 person-years. This cross-sectional incidence was lower than 18 per 100 person-years reported by the Drug User Intervention Trial (DUIT) prospective study (2002-2004) to study HCV seroconversion risk among YPWID in five major cities including NYC, which recruited street sample of 18-30-year-old PWID and a 19.5 per 100 person-years older PWID drug treatment sample (mean age 41.2) in NYC during 2006-2013 (39). The data presented in this manuscript, in combination with results from earlier studies, suggest a possible reduction in HCV prevalence and incidence among YPWID in NYC. This reduction in HCV exposure might be the result of a combination of long sustained harm reduction efforts including the public funding of needle exchange programs (since 1992), wide availability of medically assisted treatment (mainly methadone or buprenorphine) (56), and HCV treatment). As we reported in a previous paper (46), 55% of YPWID in NYC reported using needle exchange programs (NEP) in the past year; 85% ever engaged in substance use treatment (SUD); and, among those aware of their HCV-positive status, 28% had initiated HCV treatment. Modeling studies suggest that these combination interventions (NEP, SUD and HCV Treatment) can achieve reductions in HCV transmission among PWID (57). It is worth noting that the overall prevalence we report here for YPWID (i.e., 30.4%) is less than half of the 67% HCV prevalence reported for older PWID during the 2006-2013 period (39). However, this hypothesized effect from the “harm reduction protective factor” may have a limited impact. The present study reports a higher HCV prevalence, as years of IDU increase with those having injected 8 or more years presenting a prevalence (i.e., 59.7%) similar to the reported for older PWIDs in NYC by Eckhardt et al. (i.e., 63%) (17) and Jordan et al. (i.e., 67%) (39). Despite this high HCV prevalence, according to several studies, a considerable percentage (between 30–40%) remain unexposed to the HCV virus in this high risk environment, even with many years of injection.

Variables significantly associated with HCV antibody positivity among YPWID in the present study are similar to those reported by other studies. HCV positive status was independently correlated with drug injection-related variables such as having shared cookers with two or more people and IDU for 4 or more years, consistent with multiple studies demonstrating associations between HCV-positive serostatus and the sharing of injection paraphernalia and duration of IDU (5, 7, 16, 17, 58). HCV positive status was also independently correlated with structural factors such as lifetime homelessness and having been incarcerated two or more times, consistent with other studies on HCV with homelessness (16, 17, 54, 59); and those reporting high prevalence of HCV among incarcerated populations (20–22).

Collectively, these results suggest that harm reduction efforts in NYC have an overall positive impact on reducing HCV prevalence among YPWID, even as IDU risks and structural factors remain areas of key public health concern. If eliminating HCV among youth and young adults is the end goal, both IDU risk and structural factors will need to be addressed at the same time.

In an effort to better understand how these risk factors interact with one another and what policies could be effective for reducing HCV infections, we utilized a qualitative system dynamics (SD) stock and flow diagram to capture and visualize potential pathways and outcomes. The qualitative model can then inform the computational SD model in the future which requires data collection, formulation, parametrization, and calibration of causal links and parameters of the model. Using the qualitative model we can identify the kind of empirical data that is needed to validate the future computational model.

The present study provides an example of how epidemiologic analysis and statistical associations, alongside the knowledge from the extant research literature, can inform the development of a qualitative SD model to illustrate how multiple factors at different levels (e.g., structural and behavioral) interact to increase or decrease risk for and rates of HCV infection among YPWID. Our exploratory SD model visualizes the dynamic interaction among *structural factors* (e.g., drug treatment, HCV treatment, harm reduction, criminal justice, and homelessness), *injection network* characteristics (e.g., knowing opioid users older than 29), and *injection trajectory* features among YPWID (e.g., transition to IDU and duration of IDU). This model facilitates the process of hypothesizing various pathways to HCV transmission, as well as the possible impact of potential policy changes affecting upstream factors (e.g., housing assistance to decrease youth homelessness, increased availability, and uptake of HCV treatment among YPWID to reduce community HCV prevalence, reducing incarceration).

As more YPWID who are HCV-infected receive HCV treatment, an increasing number of them will clear the virus and flow back into the stock of “HCV Susceptible—YPWID” with multiple years of IDU (Figure 1). Thus, under this scenario, as the overall HCV prevalence decreases, and R1—*HCV Spread among YPWID with*<*1 Year of IDU* and R2—*HCV Spread among YPWID with* ≥*1 Year of IDU* feedback loops will become virtuous and help slow the spread of HCV. YPWID who are HCV positive, however, may continue to inject drugs—i.e., the baseline HCV prevalence within the young injection networks will increase, leading to a higher likelihood of infection per injection risk event among the uninfected. As a result, the feedback loops R1 and R2 could lead to an increasingly rapid spread of HCV among PWID. The feedback loop R2 has a stronger impact on the spread of HCV than R1 due to the higher HCV incidence among YPWID with ≥1 year IDU, indicating a need to prevent recently initiated YPWID from engaging in long-term IDU. Prevention efforts and HCV treatment focused on recently initiated YPWID could help prevent and eventually eliminate HCV in this at-risk-group (46, 50).

Homelessness is another risk factor that has been shown to be associated with greater vulnerability to HCV infection for a variety of reasons, such as the increased likelihood of injecting drugs in public spaces and limited ability to store sterile injection equipment (23–25). Thus, homelessness can make the reinforcing loops R1 and R2 become even more vicious, thereby accelerating the spread of HCV. Since people who inject drugs for multiple years may be at increased risk of homelessness due to loss of social support and the economic burden of sustaining drug use, there is an even greater urgency to provide housing and prevent transition and continuation of IDU (e.g., by expanding and facilitating access to evidence-based drug treatment among PWID).

SD can also facilitate the identification of potential policy leverage points. In the present qualitative stock and flow model we identify hypothetical policies, or structural interventions (e.g., housing for YPWID, reducing incarceration, and increased availability of harm reduction services), that could counteract the multiplying effects of risk variables (e.g., homelessness, incarceration, injection equipment sharing). For example, by expanding harm reduction services such as syringe service programs, the risk of exposure to HCV through repeated sharing of needles and cookers could be reduced. In addition, in order to break the loop of contagion, strategies and interventions could focus on preventing young people who use opioids from transitioning to IDU by providing medication for opioid use disorder (MOUD) before they begin to inject drugs or early in their injection careers. Furthermore, scaling up of treatment for HCV-infected YPWID (causing them to exit the model's two lower stocks) could decrease the baseline HCV prevalence in this population [and potentially eliminate HCV in some injection networks of YPWID (47)], thereby further reducing the outward transmission of HCV.

The SD approach extends our ability to study a complex problem by using a non-linear and operational thinking methodology that augments the traditional, linear data analysis and stepwise approach (43). SD modeling could contribute to a better understanding of interactions between variables, highlighting structural components beyond individual behaviors, and facilitating the development of comprehensive prevention policies that could include measures to address key structural factors. The qualitative SD model presented in this study could serve as the basis for developing a simulation model of HCV transmission among YPWID by mathematically quantifying the links. The simulation model can also be used to capture how the surging trend of methamphetamine and polysubstance use among individuals who use opioids, may accelerate HCV risk due to high frequency of IDU in a given period of time. SD modeling can help us to better understand how these dynamics and trends may impact individual trajectories of drug use and transitions to heroin use and IDU over time, and spread of HCV among YPWID.

Once the model is validated with historical time series data (potential next step of our effort), the model could test what-if simulation scenarios and evaluate the effectiveness of different intervention and policy strategies. In future research, the use of SD modeling could facilitate the generation of novel hypotheses and *in silico* evaluation of the combined effects of various intervention strategies over the short and long term, as well as the identification of potential unintended consequences.

## Limitations

This study has several limitations. First, given that it is a cross-sectional study, the data only provide a snapshot of the prevalence of HCV among YPWID in NYC and were not able to establish causation. The method used to calculate HCV incidence by years of IDU is limited in that we do not know whether the participant was continuously injecting drugs from the time of their first injection to the date of the interview. Also, assuming that HCV exposure occurred at the midpoint of their IDU years represents a gross estimation. However, despite these limitations, we opted to use this estimate because there is no other better way to ascertain time of HCV exposure in this cross-sectional community sample. To the best of our knowledge there is no current or recent longitudinal study that reports on HCV incidence among YPWID in NYC, hence, although imperfect, this is the best approximation we have at the moment. Also, given that this calculation method has been used by other researchers, it allows comparison across samples from the various studies in the literature. The present study focuses exclusively on young adults in NYC who inject drugs. The results, therefore, may not be generalizable to drug users of other ages or in other areas, particularly those residing in non-urban areas. The use of a non-random recruitment strategy—Respondent-Driven Sampling—may have also introduced bias into the sample that limits the generalizability of the findings such as the limited representation of Blacks in the sample. Lastly, the participants' ability to recall past exposures makes this study susceptible to recall bias.

## Conclusions

YPWID in NYC remain at high risk for HCV and thus, represents a key population in need of targeted prevention efforts if we are to eliminate HCV altogether in this group. Although this study relies heavily on qualitative analysis of the potential influence of various structural interventions, and further confirmations are required with more empirical tests (e.g., threshold analysis or sensitivity analyses), present study results suggest that harm reduction efforts in NYC may have had a positive impact overall on reducing HCV incidence and prevalence among YPWID in the city; although, injection risks (i.e., sharing cookers and long-term IDU) and structural factors (i.e., homelessness and incarceration) remain concerns for this vulnerable population. Study results also illustrate how epidemiological data can be used to help inform the development of SD models that can identify non-linearities and feedback loop structures typically missed by conventional statistical models. The combined epidemiologic and SD approach could provide important insights and contribute to an improved understanding of how multilevel risk factors interact with one another and what policies should be most effective and implemented to reduce HCV infections in YPWID.

### Policy and practice implications

Results from the present study point to the needs for further research and intervention development in this field. For example, further research could investigate the HCV incidence in a longitudinal cohort of YPWID and the casual relationship between HCV positive status and some of the variables reported among young opioid users (e.g., incarceration, homelessness, and sharing practices). Interventions that target the homeless population and those involved with criminal justice may also be important and an efficient way to identify YPWID at risk for HCV and to treat them if they are HCV-positive, especially given that HCV (for certain genotypes) is now potentially curable with the latest antiviral drugs (60). SD modeling offers tremendous value for informing policies and interventions that can either prevent IDU altogether or get youth and young adults who are already infected into earlier treatment for both HCV and addiction. Since uninfected YPWID are connected to young opioid users who are often infected, these connections also provide a pathway for the transmission of HCV. Harm reduction efforts should teach YPWID skills and strategies that can help them avoid long-term risk of HCV. In turn, healthy protective behaviors can be taught and implemented to strategically spread among YPWID's injection networks.

## Data availability statement

The datasets used and/or analyzed during the current study are available from the corresponding author on reasonable request.

## Ethics statement

The studies involving human participants were reviewed and approved by NDRI. The patients/participants provided their written informed consent to participate in this study.

## Author contributions

PM-G contributed to the conceptualization of the manuscript, prepared the original draft, and reviewed and edited the manuscript. NS contributed to the conceptualization and methodology of the manuscript, prepared the original draft, and reviewed and edited the manuscript. HG reviewed and edited the manuscript. CC prepared the original draft and reviewed and edited the manuscript. KJ prepared the original draft. BE reviewed and edited the manuscript. CF curated the data and performed the formal analysis. TH reviewed and edited the manuscript. All authors read and approved the final version of the published article.

## Funding

This research was supported by the National Institutes of Health (NIH)/National Institute on Drug Abuse (NIDA) Grants R01DA035146, R01DA041501, and K01 DA048172. The contents in this article are the sole responsibility of the authors and do not necessarily reflect the official views of NIH, NIDA, the authors' affiliations, or any organizations, agencies mentioned in the text.

## Conflict of interest

The authors declare that the research was conducted in the absence of any commercial or financial relationships that could be construed as a potential conflict of interest.

## Publisher's note

All claims expressed in this article are solely those of the authors and do not necessarily represent those of their affiliated organizations, or those of the publisher, the editors and the reviewers. Any product that may be evaluated in this article, or claim that may be made by its manufacturer, is not guaranteed or endorsed by the publisher.

## Abbreviations

HCV: Hepatitis C virus
SD: System dynamics
YPWID: Young people who inject drugs
NYC: New York City
IDU: Injection drug use
PO: Prescription opioid
OUD: Opioid use disorder
RDS: Respondent-driven sampling
Ab+: Antibody positive
SRO: Single room occupancy hotel
PYI: Person-years of injection
HIV: Human immunodeficiency virus
MOUD: Medication for opioid use disorder.
